# Monoclonal Antibodies against Zika Virus NS1 Protein Confer Protection via Fc**γ** Receptor-Dependent and -Independent Pathways

**DOI:** 10.1128/mBio.03179-20

**Published:** 2021-02-09

**Authors:** Lei Yu, Xinglong Liu, Xianmiao Ye, Wan Su, Xiaoyan Zhang, Weiqi Deng, Jia Luo, Mengrong Xiang, Wenjing Guo, Shengnan Zhang, Wei Xu, Qihong Yan, Qian Wang, Yilan Cui, Caixia Wu, Wenjing Guo, Xuefeng Niu, Fuchun Zhang, Chunliang Lei, Linbing Qu, Ling Chen, Liqiang Feng

**Affiliations:** a Guangzhou Eighth People's Hospital, Guangzhou Medical University, Guangzhou, China; b State Key Laboratories of Respiratory Diseases, Guangdong Provincial Key Laboratory of Computational Biomedicine, Guangzhou Institutes of Biomedicine and Health, Chinese Academy of Sciences, Guangzhou, China; c School of Biomedical Sciences, Huaqiao University, Quanzhou, China; d Guangzhou Regenerative Medicine and Health Guangdong Laboratory, Guangzhou, China; e University of Chinese Academy of Sciences, Beijing, China; McMaster University

**Keywords:** Zika virus, infection, monoclonal antibody, nonstructural protein 1, neurological disease, mechanism

## Abstract

Zika virus (ZIKV) infection during pregnancy causes congenital defects such as fetal microcephaly. Monoclonal antibodies (MAbs) against the nonstructural protein 1 (NS1) have the potential to suppress ZIKV pathogenicity without enhancement of disease, but the pathways through which they confer protection remain obscure. Here, we report two types of NS1-targeted human MAbs that inhibit ZIKV infection through distinct mechanisms. MAbs 3G2 and 4B8 show a better efficacy than MAb 4F10 in suppressing ZIKV infection in C57BL/6 neonatal mice. Unlike MAb 4F10 that mainly triggers antibody-dependent cell-mediated cytotoxicity (ADCC), MAbs 3G2 and 4B8 not only trigger ADCC but inhibit ZIKV infection without Fcγ receptor-bearing effector cells, possibly at postentry stages. Destroying the Fc-mediated effector function of MAbs 3G2 and 4B8 reduces but does not abolish their protective effects, whereas destroying the effector function of MAb 4F10 eliminates the protective effects, suggesting that MAbs 3G2 and 4B8 engage both Fcγ receptor-dependent and -independent pathways. Further analysis reveals that MAbs 3G2 and 4B8 target the N-terminal region of NS1 protein, whereas MAb 4F10 targets the C-terminal region, implying that the protective efficacy of an NS1-targeted MAb may be associated with its epitope recognition. Our results illustrate that NS1-targeted MAbs have multifaceted protective effects and provide insights for the development of NS1-based vaccines and therapeutics.

## Abstract (precis)

Zika virus (ZIKV) is a mosquito-borne flavivirus that has been linked to congenital microcephaly during recent epidemics. No licensed antiviral drug or vaccine is available.

## INTRODUCTION

Zika virus (ZIKV) is a single-stranded positive-sense RNA virus belonging to the *Flaviviridae* family. ZIKV is phylogenetically close to several other flaviviruses, including dengue virus (DENV) and West Nile virus (WNV) (1). Historically, ZIKV usually causes mild or self-limited infection. However, during the recent epidemics in the Americas and Southeast Asia, ZIKV infection has been linked to severe neurological diseases such as microcephaly in newborns and Guillain-Barré syndrome in adults (1, 2). The continuous circulation of ZIKV poses a great threat to global public health. Currently, no vaccine or therapeutic agent against ZIKV is available.

ZIKV genome encodes a large polyprotein that is cleaved into three structural proteins (capsid [C], premembrane or membrane [prM/M], and envelop [E]) and seven nonstructural proteins (NS1, NS2A/B, NS3, NS4A/B, and NS5). E protein mediates virus entry and is the primary target of neutralizing antibodies. Vaccine candidates currently in preclinical and clinical trials mainly target E protein (3). E-targeted neutralizing antibodies show great potency in preventing ZIKV infection in animal models (4). However, a major concern for E-targeted vaccines or antibodies is the associated antibody-dependent enhancement (ADE) of infection (5–8). An E-targeted antibody against one flavivirus may enhance the entry of the same virus or another flavivirus into cells bearing Fcγ receptor (FcγR) under a subneutralizing condition, thereby enhancing disease severity (5, 7). DENV or WNV convalescent plasma can enhance ZIKV infection and disease in mouse models (4, 5, 8–10). A prior ZIKV infection increases the risk of severe dengue disease in pediatric cohorts (11). Thus, protective antibodies without ADE effects are desirable for the prevention and treatment of ZIKV infection.

Flaviviral NS1 is traditionally not considered a target of choice for vaccines or therapeutic antibodies due to its absence in viral particles (12). After translation and modification, NS1 is rapidly dimerized, transported to the plasma membrane, and secreted as a lipophilic hexamer (12). NS1 plays critical roles in flaviviral replication, pathogenesis, and immune evasion. DENV NS1 participates in viral replication and release (13). It also elicits autoantibodies and causes damage to endothelial cells (14). Secreted DENV NS1 not only disrupts the integrity of the endothelium and facilitates vascular leakage (15) but also activates monocytes or macrophages and triggers a “cytokine storm” (16, 17). Importantly, NS1 antigenemia is associated with the severity of dengue disease in human (18, 19). Emerging evidence shows that ZIKV NS1 can induce hyperpermeability in the umbilical vein and in brain endothelial cells, which may promote ZIKV spread to fetal brain (20). ZIKV NS1 has been shown to disrupt placental glycosaminoglycans, resulting in increased permeability of human placentas (21). ZIKV NS1 also triggers endothelial barrier dysfunction and impacts the junctional integrity of human brain microvascular endothelial cells (22). NS1 antigenemia in infected hosts enhances the acquisition of ZIKV by its mosquito vectors, which may facilitate the transmission of ZIKV during recent epidemics (23, 24).

An NS1-targeted antibody may suppress the pathogenic effects of NS1 without causing ADE of infection (25, 26). DENV NS1-targeted antibodies, either induced by immunization or passively inoculated, alleviate DENV symptoms (15). It has been shown that immunization with NS1-based vaccines confer protection against ZIKV in both adult and neonatal mice (8, 25, 27–29). Recently, several monoclonal antibodies (MAbs) against ZIKV NS1 have been isolated and shown to confer protection in STAT2-knockout adult mice (26). However, animals treated with these MAbs still experienced transient loss of body mass, and a proportion of them even died (26). It is unclear whether NS1-targeted MAbs are able to protect neonates from ZIKV-associated neurological disease. An antibody with protective effects in neonates may have significant clinical benefits, because pregnant women are most susceptible to ZIKV infection and their fetuses suffer the most devastating consequences (30). Moreover, the pathways through which NS1-targeted MAbs confer protection are not fully understood. It is thus important to develop NS1-targeted MAbs with protective effects in neonates and to delineate their protection mechanisms.

Here, we investigate the protective effects of three NS1-targeted human MAbs against ZIKV-associated diseases using a C57BL/6 neonatal mouse model. We generated Fc-mutated MAb variants that lose FcγR-binding activity and explored the pathways through which these MAbs confer protection. We also analyzed the regions on NS1 that are recognized by these MAbs.

## RESULTS

### Multiple-dose inoculation with NS1-targeted MAbs suppresses ZIKV infection and pathogenicity in C57BL/6 neonatal mice.

**FIG 1:**
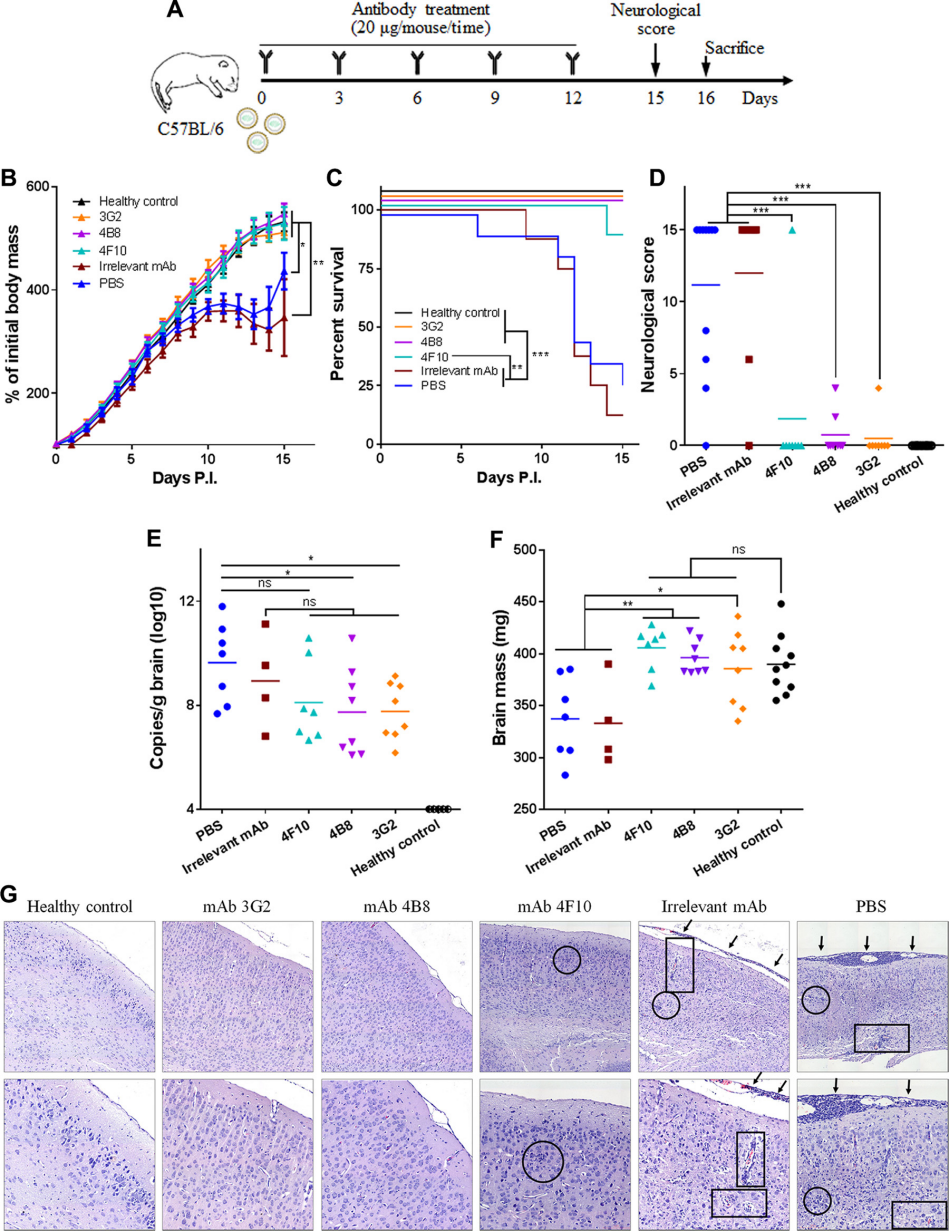
Multiple-dose inoculation with NS1-targeted MAbs suppresses ZIKV infection and the associated disease in C57BL/6 neonatal mice. (A) Schematic diagram of ZIKV challenge and MAb treatment in C57BL/6 neonatal mice. The pups were challenged with 1.2 × 10^3^ PFU ZIKV GZ02 strain. Unchallenged pups were used as healthy controls. (B) Body masses of ZIKV-challenged pups treated with or without MAbs. (C) Survival curves of ZIKV-challenged pups treated with or without MAbs. (D) Neurological symptom scores of ZIKV-challenged pups with or without MAb treatment. (E) Genome copy numbers of ZIKV detected in neonatal brains at 16 days postchallenge. (F) Masses of mouse brains at 16 days postchallenge. (G) Histological analyses of brain tissues at 16 days postchallenge. One representative image from each mouse group is shown. The magnification is ×100 (top) or ×200 (bottom). Arrows, inflammatory cell infiltration in the meninges; circles, glial nodules; rectangles, lymphocytic infiltration in the cerebrum tissue. Data are representative results from three independent experiments. Data in panel B are expressed as the means ± standard deviations (SDs). Differences in panels B, D, E, F, and G were assessed by one-way analysis of variance (ANOVA) and Tukey’s multiple-comparison test (healthy control, *n* = 10; 3G2, 4B8, 4F10, irrelevant MAb, *n* = 8; PBS, *n* = 11). Comparison of the survival rates in panel C was performed using the log rank test. *, *P* < 0.05; **, *P* < 0.01; ***, *P* < 0.001; ns, no significance.

We have isolated a series of NS1-targeted MAbs (3G2, 4B8, and 4F10) from ZIKV convalescent patients (31). To assess if these MAbs have protective effects, we used a ZIKV-infected C57BL/6 neonatal mouse model (29, 32). C57BL/6 neonatal mice were infected with the ZIKV GZ02 strain at 1.2 × 10^3^ PFU per mouse by intraperitoneal (i.p.) injection. Our preliminary data showed that at this dose, more than 75% of C57BL/6 neonates developed severe diseases or died. Because the level of NS1-targeted MAbs peaked at 24 h postinoculation in C57BL/6 neonatal mice and gradually decreased thereafter (see Fig. S1 in the supplemental material), we treated the infected neonates by i.p. inoculation with each MAb (20 μg per mouse) on days 0, 3, 6, 9, and 12 after challenge (Fig. 1A). That is, each animal received 100 μg MAb in total. The infected neonates receiving a MAb against Marburg virus (MR78) or phosphate-buffered saline (PBS) were used as an irrelevant control or an untreated control, respectively.

10.1128/mBio.03179-20.1 FIG S1 Kinetics of NS1-targeted MAbs in neonatal C57BL/6 mice. Mice were i.p. inoculated with each MAb at 20 μg/mouse. On day 0, 1, 3, 5, or 7, the mice were sacrificed and homogenized. The homogenates were collected and subjected to ELISA using ZIKV NS1 protein. The OD values at 450 nm at 1:5 are shown. Data are representative results from two independent experiments and expressed as the means ± SDs. Download FIG S1, TIF file, 1.6 MB. Copyright © 2021 Yu et al. 2021 Yu et al. This content is distributed under the terms of the Creative Commons Attribution 4.0 International license.

All the mice receiving NS1-targeted MAbs exhibited a similar growth curve as healthy control mice, whereas the mice receiving the irrelevant MAb or PBS showed severe ZIKV-caused growth delay (Fig. 1B). In the MAb 3G2-treated group, with the exception of one mouse that showed mild weakness in the hind limbs (score, 4), all mice showed no neurological abnormality throughout the experiment (score, 0). In the MAb 4B8-treated group, two mice showed weakness in the hind limbs (score, 4 and 2) but the others had no signs of illness (score, 0). In the MAb 4F10-treated group, one mouse died (score, 15) but the others showed no significant abnormality (Fig. 1C and D). In contrast, more than 75% of the mice treated with irrelevant MAb or with PBS died within 15 days postchallenge and received clinical scores of 15 (Fig. 1C and D). Therefore, NS1-targeted MAbs effectively protect C57BL/6 neonatal mice against ZIKV lethality and neurological disorders.

We next assessed if NS1-targeted MAbs reduced ZIKV infection in the neonatal brain. MAbs 3G2 and 4B8 but not 4F10 significantly reduced viral genome copies in the brain (Fig. 1E). There were no detectable live viral particles in 6 of the 8 MAb 3G2-treated mice, 5 of the 8 MAb 4B8-treated mice, and 4 of the 7 MAb 4F10-treated mice (see Fig. S2). In contrast, more than 70% of the mice receiving irrelevant MAb or PBS had large numbers of live viral particles in the brain (Fig. S2). It should be noted that a proportion of mice receiving irrelevant MAb or PBS succumbed to ZIKV disease before sacrifice and fresh brain tissues were unavailable, which may lead to an underestimate of the viral loads in these two groups. Thus, NS1-targeted MAbs, especially 3G2 and 4B8, reduce ZIKV infection in the neonatal brain. The brain mass of mice receiving NS1-targeted MAbs was comparable to that of healthy control mice but significantly higher than that of the mice receiving irrelevant MAb or PBS (Fig. 1F), suggesting that these MAbs alleviate ZIKV-caused brain damage. Accordingly, meningeal thickening and lymphocyte infiltration were observed in the mice receiving PBS or irrelevant MAb but not in those receiving MAbs 3G2, 4B8, or 4F10 (Fig. 1G). Glial nodules were present in the cerebral cortices of the mice receiving PBS, irrelevant MAb, and even MAb 4F10 but were absent in the mice treated with MAbs 3G2 or 4B8 (Fig. 1G). Together, these data show that NS1-targeted MAbs confer protection against ZIKV infection in C57BL/6 neonatal mice, and MAbs 3G2 and 4B8 appear to be more efficacious than MAb 4F10.

10.1128/mBio.03179-20.2 FIG S2 Live viral particles in the brains of ZIKV-infected neonatal C57BL/6 mice. On day 16 after challenge, the neonatal brains of surviving pups were harvested and homogenized, and the live viral particles were determined by plaque-forming assay. Virus titers in the brains were calculated by dividing the plaque counts by the mass of homogenized brain tissues. The data points are the mean values from two technical replicates, and the proportion of pups exhibiting undetectable live virions is marked. Differences among groups were assessed by one-way ANOVA and Tukey’s multiple-comparison test. **, *P* < 0.01; ns, no significance. Download FIG S2, TIF file, 1.8 MB. Copyright © 2021 Yu et al. 2021 Yu et al. This content is distributed under the terms of the Creative Commons Attribution 4.0 International license.

### Single-dose inoculation with NS1-targeted MAbs confers partial protection against ZIKV infection in C57BL/6 neonatal mice.

**FIG 2:**
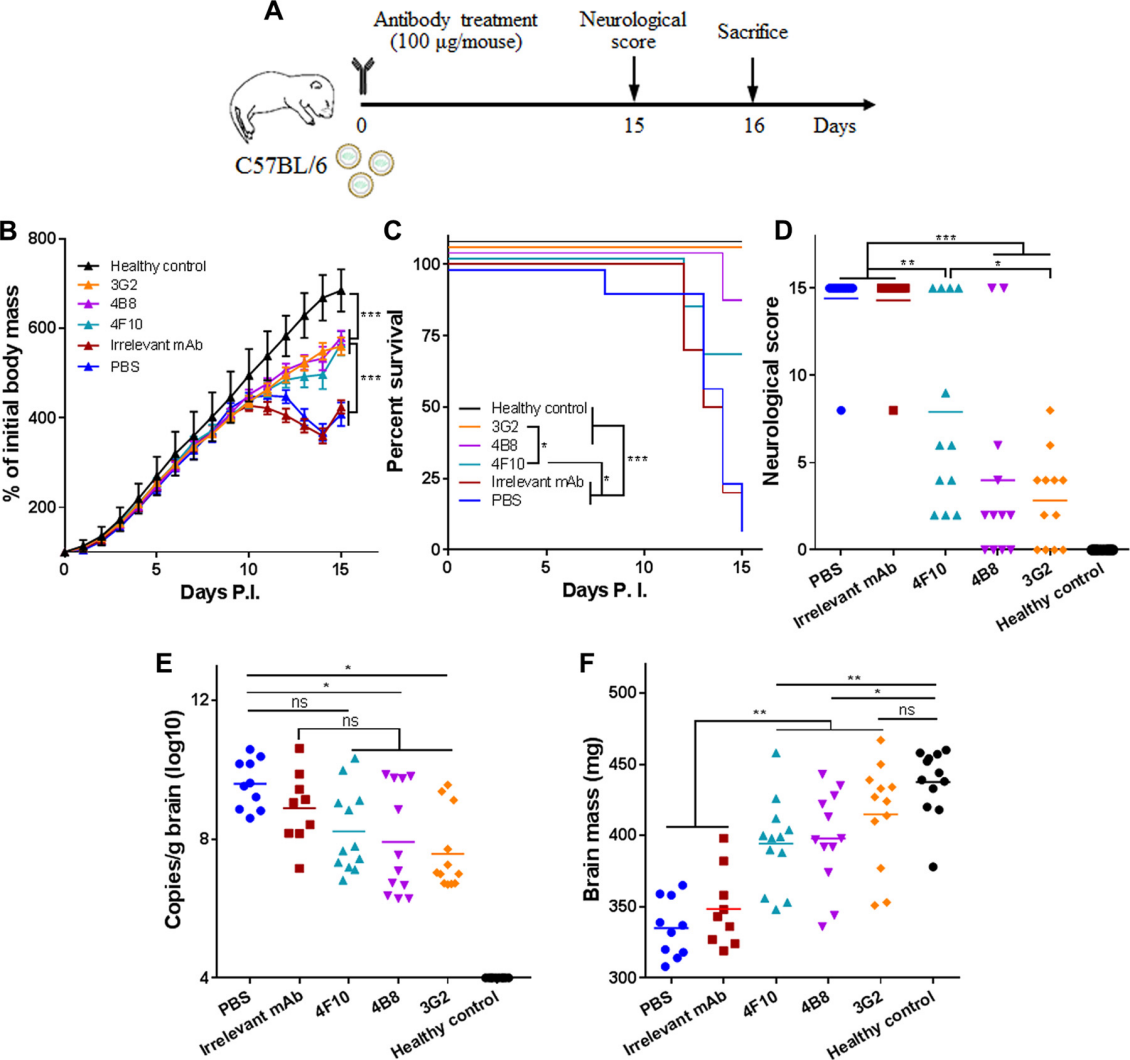
Single-dose inoculation with NS1-targeted MAbs suppresses ZIKV-associated lethality and neurological disorders in C57BL/6 neonatal mice. (A) Schematic diagram of ZIKV challenge and MAb treatment in neonatal C57BL/6 mice. The pups were challenged with 1.2 × 10^3^ PFU ZIKV GZ02 strain. Unchallenged pups were used as healthy controls. (B) Body masses of ZIKV-challenged pups treated with or without MAbs. (C) Survival curves of ZIKV-challenged pups treated with or without MAbs. (D) Neurological symptom scores of ZIKV-challenged pups with or without MAb treatment. (E) Genome copy numbers of ZIKV detected in neonatal brains at 16 days postchallenge. (F) Masses of mouse brains at 16 days postchallenge. Data are representative results from two independent experiments. Data in panel B are expressed as the means ± SDs. Differences in panels B, D, E, and F were assessed by one-way ANOVA and Tukey’s multiple-comparison test (healthy control, 3G2, 4B8, 4F10, PBS, *n* = 12; irrelevant MAb, *n* = 11). Comparison of the survival rates in panel C was performed using the log rank test. *, *P* < 0.05; **, *P* < 0.01; ***, *P* < 0.001; ns, no significance.

To assess if single-dose inoculation with these MAbs still has any protective effects, C57BL/6 neonatal mice were infected with ZIKV at 1.2 × 10^3^ PFU per mouse via i.p. injection and then were given a single inoculation of NS1-targeted MAbs or MR78 (100 μg each MAb per mouse) or PBS at 2 h postinfection (Fig. 2A). Growth delay was partially alleviated in the mice receiving NS1-targeted MAbs compared to that in mice receiving irrelevant MAb or PBS, and the alleviation was less efficient than that observed with the multiple-dose strategy (Fig. 2B). MAbs 3G2 and 4B8 greatly improved the survival rate. MAb 4F10 also improved the survival rate, but the potency was lower than that of MAb 3G2 (Fig. 2C). All the NS1-targeted MAbs effectively suppressed neurological symptoms, and MAb 3G2 had the greatest potency (Fig. 2D). MAbs 3G2 and 4B8 but not MAb 4F10 were able to reduce the viral loads in the neonatal brain (Fig. 2E). Notably, MAb 3G2 protected against ZIKV-caused delay of brain growth. MAbs 4B8 and 4F10 also had this effect, but were not as good as MAb 3G2 (Fig. 2F). Thus, NS1-targeted MAbs, especially MAb 3G2, still have protective effects in the single-dose regimen, but an ideal strategy is the multiple-dose inoculation.

### All three MAbs mediate ADCC killing of NS1-expressing cells, but only MAbs 3G2 and 4B8 inhibit ZIKV infection at postentry stages without effector cells.

**FIG 3:**
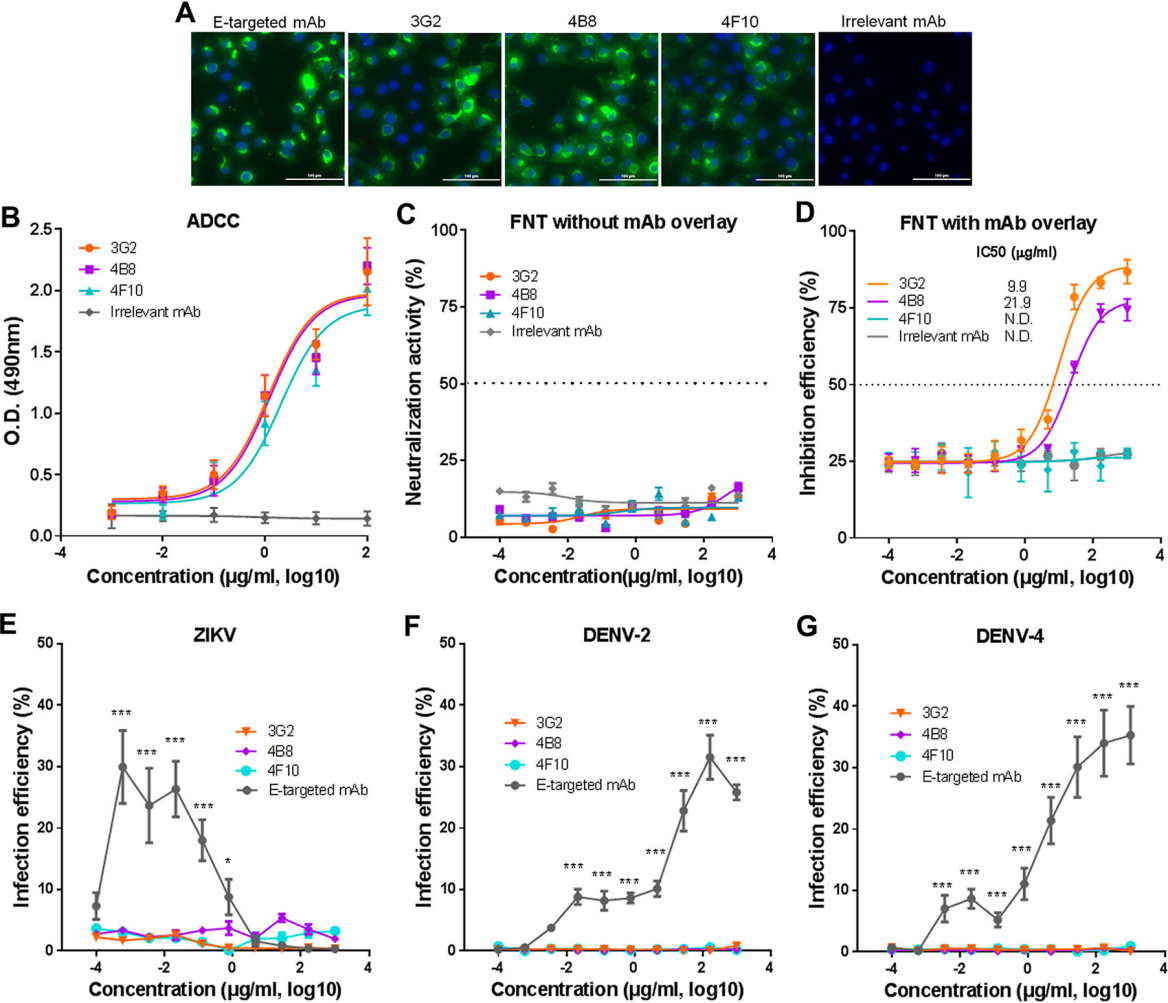
All NS1-targeted MAbs mediate ADCC activity and do not cause ADE of infection, but only MAbs 3G2 and 4B8 directly inhibit ZIKV infection in Vero cells. (A) Immunofluorescence analysis of ZIKV-infected Vero cells with MAbs 3G2, 4B8, and 4F10, an irrelevant MAb, and an E-targeted MAb (8D10). Scale bars, 100 nm. (B) ADCC activity mediated by NS1-targeted MAbs. The release of lactate dehydrogenase (LDH) was measured as a surrogate marker for target cell lysis and cell death. (C) Neutralizing activity of NS1-targeted MAbs against ZIKV GZ02 strain detected by FNT without MAb overlay. (D) Inhibition efficiency of NS1-targeted MAbs against ZIKV GZ02 strain detected by FNT with MAb overlay. (E) Infection efficiency of ZIKV in K562 cells in the presence of NS1-targeted or E-targeted MAbs. (F) Infection efficiency of DENV-2 in K562 cells in the presence of NS1-targeted or E-targeted MAbs. (G) Infection efficiency of DENV-4 in K562 cells in the presence of NS1-targeted or E-targeted MAbs. Data are representative results from three independent experiments and expressed as the means ± SDs. Each data point represents three technical replicates. Differences in panels E, F, and G were assessed using one-way ANOVA and Tukey’s multiple-comparison test (*n* = 3). *, *P* < 0.05; ***, *P* < 0.001; N.D., not detectable.

It has been shown that ZIKV NS1-targeted MAbs can exert protective effects through triggering antibody-dependent cell-mediated cytotoxicity (ADCC) (26). We thus tested if the different protective effects of NS1-targeted MAbs resulted from their capability to mediate ADCC. All three MAbs effectively recognized the NS1 in Vero cells that were infected with the ZIKV GZ02 strain (Fig. 3A). At least a proportion of cells expressed NS1 on the cellular surface, similar to E protein (Fig. 3A). We then performed an ADCC assay using Vero cells that ectopically expressed GZ02 NS1 as target cells and using human peripheral blood mononuclear cells (PBMCs) as effector cells. All the MAbs mediated cytolysis of NS1-expressing cells in a dose-dependent manner and with a comparable efficacy (Fig. 3B), suggesting that these MAbs have similar capabilities to mediate ADCC.

Our previous results showed that mouse immune sera containing NS1-targeted antibodies inhibited ZIKV infection in the absence of effector cells (24). We thus hypothesized that NS1-targeted MAbs might also inhibit ZIKV infection without effector cells. To test this hypothesis, we performed a flow cytometry-based neutralization test (FNT) with or without MAb overlay (29). For FNT without MAb overlay, MAbs were incubated with ZIKV in cultured Vero cells for 2 h, and then the culture media were replaced to determine the effects of MAbs on the early or entry stage of infection. For FNT with MAb overlay, MAbs were incubated with ZIKV in cultured Vero cells for 4 days until detection. None of these MAbs showed neutralizing activity in FNT without MAb overlay (Fig. 3C), implying that NS1-targeted MAbs cannot block the binding or entry of ZIKV. No NS1 protein was detected on purified ZIKV virions (see Fig. S3), also supporting that NS1-targeted MAbs are unlikely to have neutralizing activity. However, in FNT with MAb overlay, MAbs 3G2 and 4B8 but not MAb 4F10 effectively inhibited ZIKV infection. MAb 3G2 (half-maximal inhibitory concentration [IC_50_] = 9.9 μg/ml) showed a somewhat higher inhibitory effect than MAb 4B8 (IC_50_ = 21.9 μg/ml) (Fig. 3D). This result suggests that a subset of NS1-targeted MAbs such as 3G2 and 4B8 can inhibit ZIKV infection without effector cells.

10.1128/mBio.03179-20.3 FIG S3 Western blot analysis of NS1 protein on purified ZIKV virions. ZIKV was propagated in Vero cells and purified by sucrose density gradient centrifugation. Viral particles were added with 1× loading buffer without β-mercaptoethanol, boiled, and subjected to SDS-PAGE. NS1 protein was labeled with an anti-Zika virus NS1 MAb B4 (cat no. ab218546, Abcam). Mock-infected Vero cells and purified ZIKV NS1 protein were examined in parallel as negative and positive controls, respectively. Lane 1, mock-infected Vero cells; lane 2: purified ZIKV virions, 2 × 10^4^ PFU; lane 3, purified ZIKV virions, 2 × 10^5^ PFU; lane 4, ZIKV NS1, 0.4 μg; lane 5, ZIKV NS1, 2 μg. Download FIG S3, TIF file, 2.4 MB. Copyright © 2021 Yu et al. 2021 Yu et al. This content is distributed under the terms of the Creative Commons Attribution 4.0 International license.

To determine the possible stages of ZIKV infection that may be affected by NS1-targeted MAbs, we performed binding and entry assays. ZIKV was preincubated with each MAb, chilled on ice, and inoculated into Vero cells. For the binding assay, the cells were thoroughly washed and immediately subjected to RNA extraction. For the entry assay, the cells were washed, resuspended, incubated at 37°C for another 2 h, and subjected to RNA extraction. None of the NS1-targeted MAbs or the irrelevant MAb showed any inhibition on viral binding or entry, whereas the E-targeted neutralizing MAb 7B3 effectively blocked viral binding and entry (see Fig. S4). This result confirms that NS1-targeted MAbs have no neutralizing activity. We next determined if NS1-targeted MAbs affected ZIKV infection at postentry stages. Vero cells were infected with ZIKV in the presence of each MAb during hours 0 to 6, 6 to 12, 12 to 18, or 18 to 24 after infection (see Fig. S5A). In our infection assay, we found that progeny virions started to be released 18 to 24 h after infection (Fig. S5B and C). When present at 0 to 6 h after infection, none of the NS1-targeted MAbs significantly affected the number of viral genome copies in the cells or the culture supernatants at 24 h after infection (Fig. S5D and H), consistent to the observation that they have no neutralizing activity (Fig. 3C and S4). However, MAbs 3G2 and 4B8 appeared to reduce viral replication and release when present 6 to 12 and 12 to 18 h after infection (Fig. S5E, F, I, and J). When present 18 to 24 h after infection, these two MAbs moderately suppressed viral replication but significantly reduced the release of progeny virions (Fig. S5G and K), revealing an inhibition of viral egress. In contrast, MAb 4F10 was similar to MR78, and both showed no inhibitory effects regardless of their overlay duration (Fig. S5D to G). Thus, MAbs 3G2 and 4B8 but not 4F10 most likely inhibit ZIKV infection at postentry stages.

10.1128/mBio.03179-20.4 FIG S4 ZIKV binding and entry in the presence of NS1-targeted MAbs. (A) Relative ZIKV binding in the presence of NS1-targeted MAbs. (B) Relative ZIKV entry in the presence of NS1-targeted MAbs. ZIKV was preincubated with each MAb or PBS, chilled on ice, and coincubated with Vero cells at 4°C for 2 h. (A) For the binding assay, the genome copies of the bound virions were measured by RT-qPCR. (B) For the entry assay, after thoroughly washing with ice-cold PBS, cells were incubated at 37°C for another 2 h, and the genome copies of internalized virions were measured by RT-qPCR. An irrelevant MAb MR78 and an E-targeted MAb 7B3 were examined in parallel as negative and positive controls, respectively. Relative viral binding (A) and viral entry (B) were calculated as the ratio of the absolute genome copies in each MAb-treated group to those in PBS-treated group. Data are representative results of three independent experiments and expressed as means ± SDs. Differences among groups were assessed by one-way ANOVA and Tukey’s multiple-comparison test (*n* = 3). ***, *P* < 0.001; ns, no significance. Download FIG S4, TIF file, 1.3 MB. Copyright © 2021 Yu et al. 2021 Yu et al. This content is distributed under the terms of the Creative Commons Attribution 4.0 International license.

10.1128/mBio.03179-20.5 FIG S5 Analysis of the impacts of NS1-targeted MAbs on different stages of ZIKV infection. (A) Schematic diagram of the infection assay in the presence of NS1-targeted MAbs at different periods after ZIKV infection. (B) Growth curves of ZIKV in Vero cells. (C) Growth curves of ZIKV in the culture supernatants. Vero cells were infected with ZIKV at 2 or 0.2 PFU per cell. At the indicated time points, the genome copies in the cells (B) and the culture supernatants (C) were assessed by RT-qPCR. Relative viral genome copies in Vero cells in the presence of each MAb during hours 0 to 6 (D), 6 to 12 (E), 12 to 18 (F), and 18 to 24 (G) after infection. Relative viral genome copies in the culture supernatants in the presence of each MAb during hours 0 to 6 (H), 6 to 12 (I), 12 to 18 (J), and 18 to 24 (K) after infection. Vero cells were infected with ZIKV at 2 PFU per cell. NS1-targeted MAbs were added at 0, 6, 12, or 18 h after infection, and the culture supernatants were replaced with fresh culture medium 6 h later. An irrelevant MAb MR78 was examined in parallel as a negative control. At 24 h after infection, ZIKV genome copies in cells (D to G) and the culture supernatants (H to K) were assessed by RT-qPCR. Relative genome copies were calculated as the ratio of absolute genome copies in each MAb-treated group to those in PBS-treated group. Data are representative results of three independent experiments and expressed as means ± SDs. Differences among groups were assessed by one-way ANOVA and Tukey’s multiple-comparison test (*n* = 3). *, *P* < 0.05; **, *P* < 0.01; ***, *P* < 0.001; ns, no significance. Download FIG S5, TIF file, 2.0 MB. Copyright © 2021 Yu et al. 2021 Yu et al. This content is distributed under the terms of the Creative Commons Attribution 4.0 International license.

Because NS1 is not present on flaviviral particles, MAbs against ZIKV NS1 are unlikely to elicit ADE of infection of either ZIKV or genetically related DENV. To test this hypothesis, we examined the infection of ZIKV and DENV in K562 cells in the presence of NS1-targeted MAbs. Unlike the E-targeted MAb 8D10 that showed significant ADE of ZIKV infection, none of the NS1-targeted MAbs enhanced ZIKV infection (Fig. 3E). MAb 8D10 also promoted the infection of DENV-2 and DENV-4, but the NS1-targeted MAbs did not show this effect at any of the tested concentrations (Fig. 3F and G). Thus, NS1-targeted MAbs do not seem to cause ADE of infection of either ZIKV or DENV.

### The LALAPG variants of MAbs 3G2 and 4B8 lost ADCC activity but retained inhibitory effects against ZIKV infection in cell cultures.

**FIG 4:**
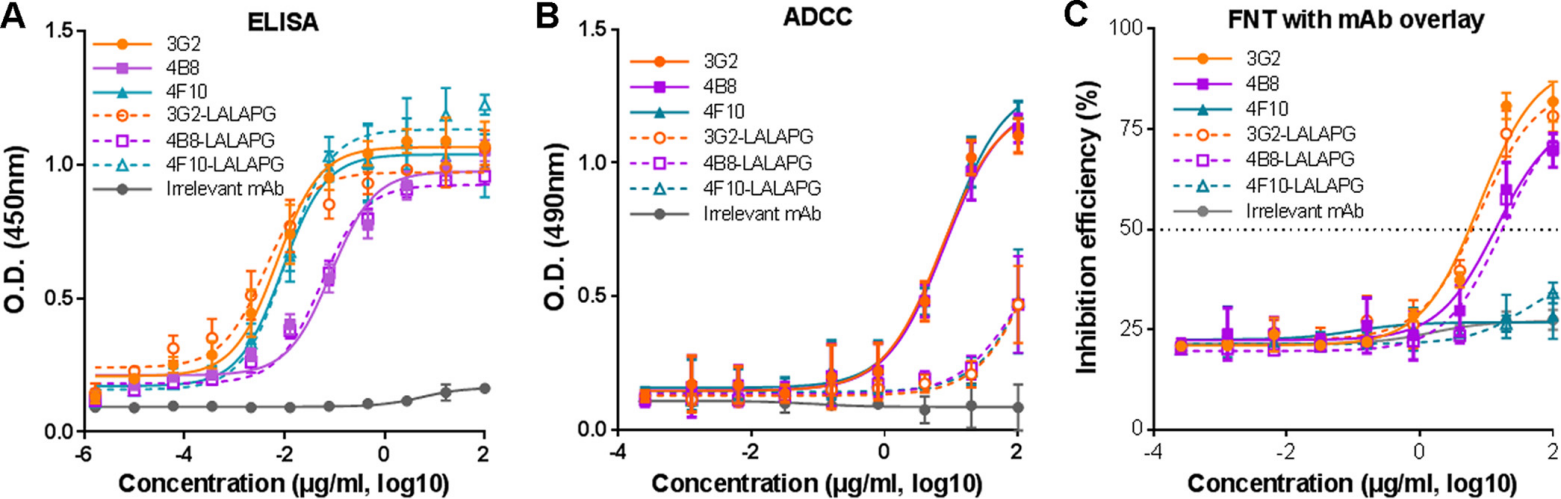
Fc mutation eliminates the ADCC activity of NS1-targeted MAbs but does not significantly affect the inhibition effects of MAbs 3G2 and 4B8. (A) Binding activity of NS1-targeted MAbs and their respective LALAPG variants to ZIKV-infected Vero cells. (B) ADCC activity mediated by NS1-targeted MAbs and the LALAPG variants. The release of lactate dehydrogenase (LDH) was measured as a surrogate marker for target cell lysis and cell death. (C) Inhibition efficiency of NS1-targeted MAbs and their respective LALAPG variants against ZIKV GZ02 strain detected by FNT without MAb overlay. Data are representative results from three independent experiments and expressed as the means ± SDs. Each data point represents three technical replicates.

To confirm the FcγR-independent inhibitory effects of NS1-targeted MAbs, we generated a variant of each MAb that harbors three amino acid substitutions (L234A, L235A, and P239G) in the Fc region, named 3G2-LALAPG, 4B8-LALAPG, and 4F10-LALAPG. These amino acid substitutions have been shown to eliminate the ADCC activity and complement binding activity of human IgG1 (33). The variants and their wild-type counterparts had comparable binding activities to ZIKV NS1 (Fig. 4A), suggesting that these substitutions do not alter the binding activity to ZIKV-infected Vero cells. All three variants showed a sharply decreased ADCC activity compared to that of the wild-type MAbs (Fig. 4B), implying that their binding capability to Fcγ receptor was greatly reduced. Notably, similar to MAbs 3G2 and 4B8, MAbs 3G2-LALAPG and 4B8-LALAPG but not 4F10-LALAPG effectively inhibited ZIKV infection in Vero cells (Fig. 4C). This result further supports that MAbs 3G2 and 4B8 but not 4F10 are able to directly inhibit ZIKV infection in addition to mediating ADCC.

### Multiple-dose inoculation of 3G2-LALAPG and 4B8-LALAPG but not 4F10-LALAPG protects against ZIKV lethality in C57BL/6 neonatal mice.

**FIG 5:**
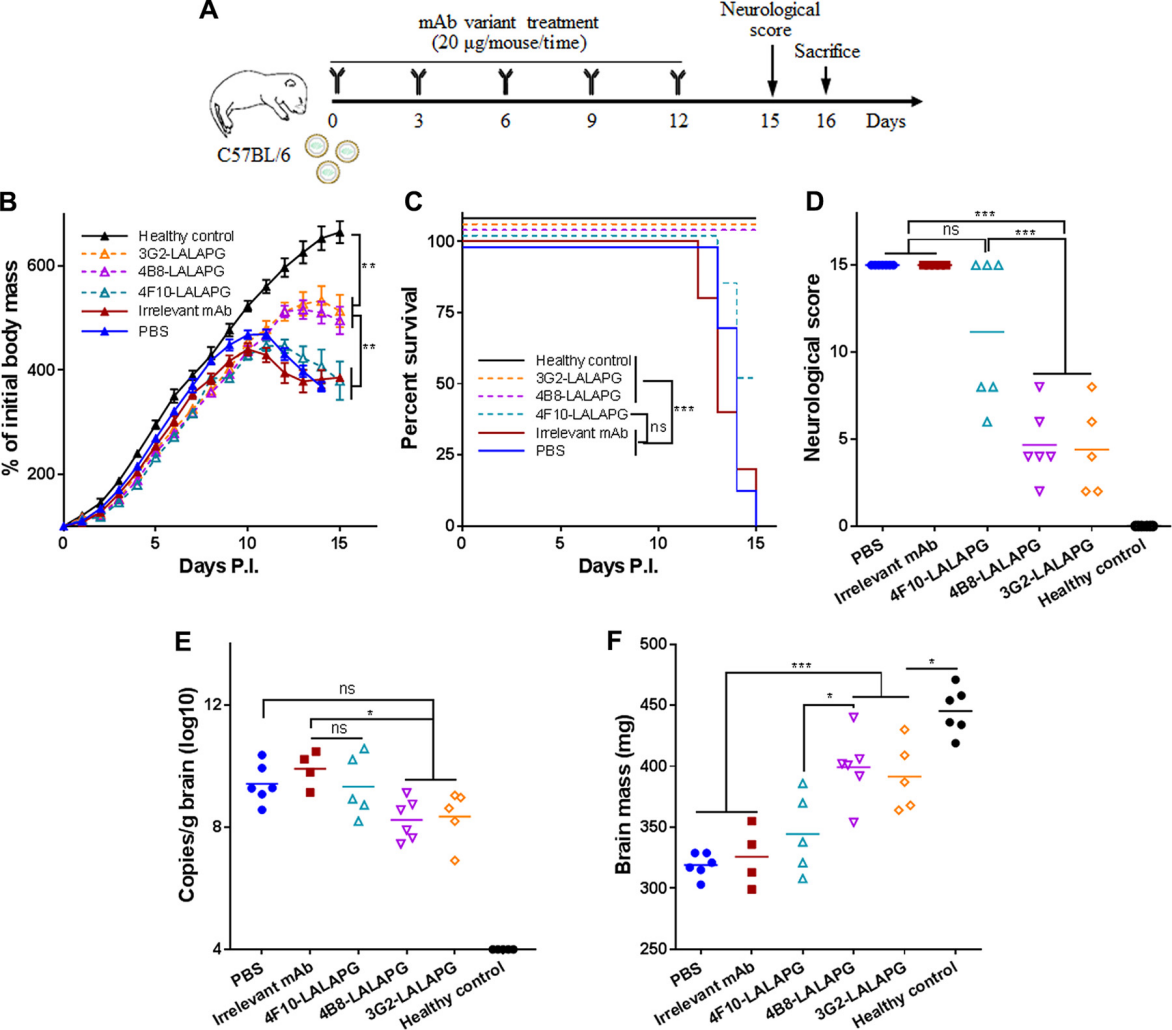
Multiple-dose inoculations with the LALAPG variants of MAbs 3G2 and 4B8 but not 4F10 protect against ZIKV lethality in C57BL/6 neonatal mice. (A) Schematic diagram of ZIKV challenge and MAb variant treatment in C57BL/6 neonatal mice. The pups were challenged with 1.2 × 10^3^ PFU ZIKV GZ02 strain. Unchallenged pups were used as healthy control. (B) Body masses of ZIKV-challenged pups treated with or without MAb variants. (C) Survival curves of ZIKV-challenged pups treated with or without MAb variants. (D) Neurological symptom scores of ZIKV-challenged pups treated with or without MAb variants. (E) Genome copy numbers of ZIKV detected in neonatal brains at 16 days postchallenge. (F) Masses of mouse brains at 16 days postchallenge. Data in panel B are expressed as the means ± SDs. Differences in panels B, D, E, F, and G were assessed by one-way ANOVA and Tukey’s multiple-comparison test (healthy control, 4B8-LALAPG, 4F10-LALAPG, *n* = 6; 3G2, irrelevant MAb, *n* = 5; PBS, *n* = 7). Comparison of the survival rates in panel C was performed using the log rank test. *, *P* < 0.05; **, *P* < 0.01; ***, *P* < 0.001; ns, no significance.

To determine if the LALAPG variants still have any protective effects *in vivo*, C57BL/6 neonatal mice were infected with ZIKV at 1.2 × 10^3^ PFU per mouse via i.p. injection and then treated with each variant or MR78 using a multiple-dose inoculation strategy (20 μg per mouse at each time point) (Fig. 5A). 3G2-LALAPG and 4B8-LALAPG but not 4F10-LALAPG partially alleviated ZIKV-caused growth delay (Fig. 5B). All the mice receiving 3G2-LALAPG or 4B8-LALAPG survived, but 50% of the mice receiving 4F10-LALAPG died (Fig. 5C). 3G2-LALAPG and 4B8-LALAPG but not 4F10-LALAPG significantly reduced ZIKV-associated neurological disorders and brain viral loads and partially reversed the growth delay of the neonatal brain (Fig. 5D to F). Because 3G2-LALAPG and 4B8-LALAPG did not completely inhibit the ZIKV-caused growth delay and brain damage as did MAbs 3G2 and 4B8, both FcγR-dependent and -independent pathways participate in the protection of MAbs 3G2 and 4B8. MAb 4F10, however, is largely dependent on Fc-mediated effector functions.

### Single-dose inoculation with 3G2-LALAPG but not 4B8-LALAPG or 4F10-LALAPG shows mild protection against ZIKV lethality in C57BL/6 neonatal mice.

**FIG 6:**
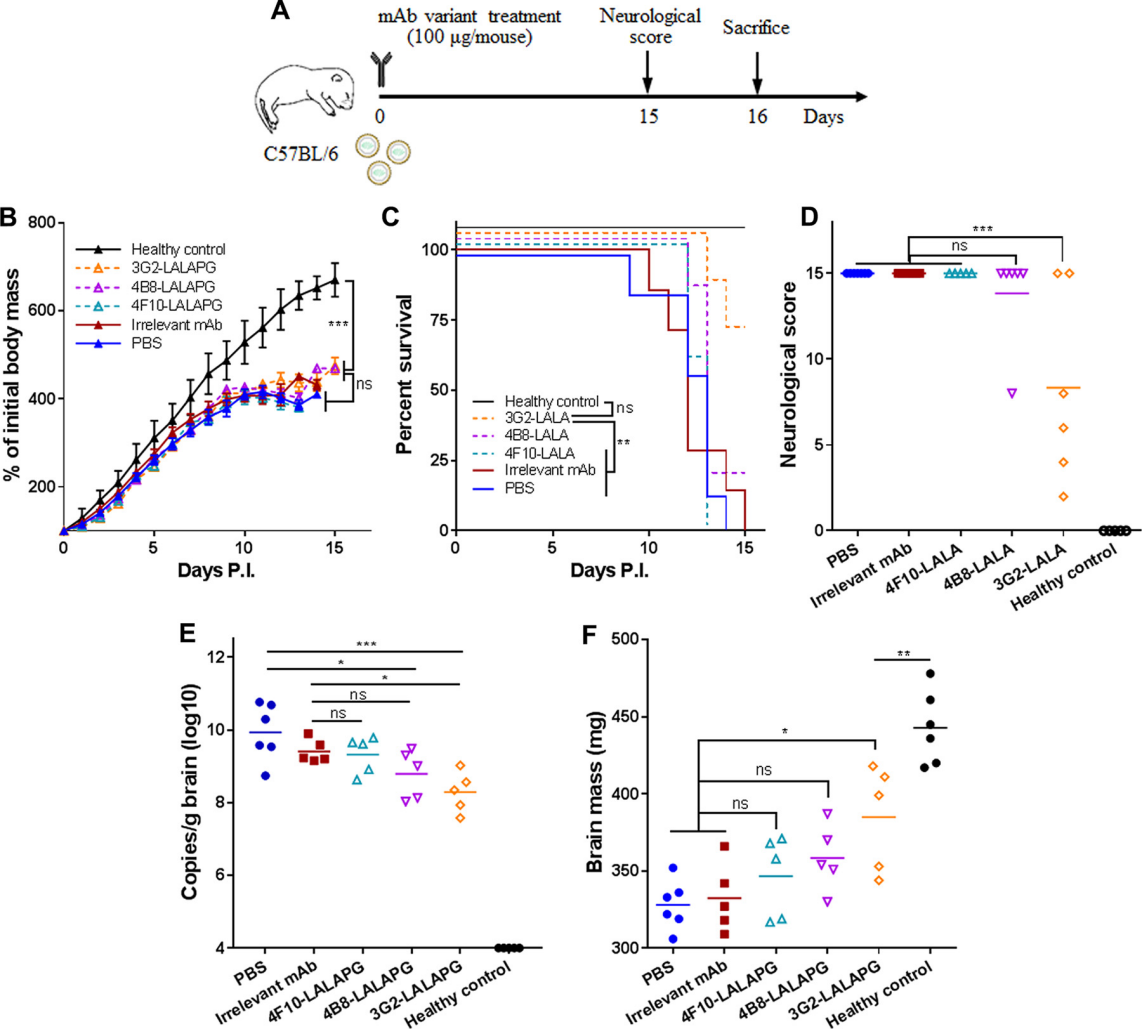
Single-dose inoculation with the variant of MAb 3G2 but not the other two variants only provides mild protection. (A) Schematic diagram of ZIKV challenge and MAb variant treatment in C57BL/6 neonatal mice. The pups were challenged with 1.2 × 10^3^ PFU ZIKV GZ02 strain. Unchallenged pups were used as healthy control. (B) Body masses of ZIKV-challenged pups treated with or without MAb variants. (C) Survival curves of ZIKV-challenged pups treated with or without MAb variants. (D) Neurological symptom scores of ZIKV-challenged pups treated with or without MAb variants. (E) Genome copy numbers of ZIKV detected in neonatal brains at 16 days postchallenge. (F) Masses of mouse brains at 16 days postchallenge. Data in panel B are expressed as the means ± SDs. Differences in panels B, D, E, and F were assessed by one-way ANOVA and Tukey’s multiple-comparison test (healthy control, 4B8-LALAPG, 4F10-LALAPG, *n* = 6; 3G2, irrelevant MAb, *n* = 5; PBS, *n* = 7). Comparison of the survival rates in panel C was performed using the log rank test. *, *P* < 0.05; **, *P* < 0.01; ***, *P* < 0.001; ns, no significance.

To determine if single-dose inoculation with LALAPG variants has any protective effects, C57BL/6 neonatal mice were infected with ZIKV at 1.2 × 10^3^ PFU per mouse and then treated with one dose of each variant (100 μg per mouse) or MR78 (Fig. 6A). In this setting, none of the variants reversed the ZIKV-caused growth delay (Fig. 6B). Only 3G2-LALAPG (not 4B8-LALAPG or 4F10-LALAPG) significantly improved the survival rate (Fig. 6C). 3G2-LALAPG also reduced the neurological disorders and brain viral loads and partially reversed the growth delay of the neonatal brain (Fig. 6D to F). Notably, the efficacy of 3G2-LALAPG in the single-dose regimen was lower than that in the multiple-dose regimen, further supporting that NS1-targeted MAbs should be given via multiple-dose administration.

### MAbs 3G2 and 4B8 recognize the N-terminal region, whereas MAb 4F10 targets the C-terminal region of NS1.

**FIG 7:**
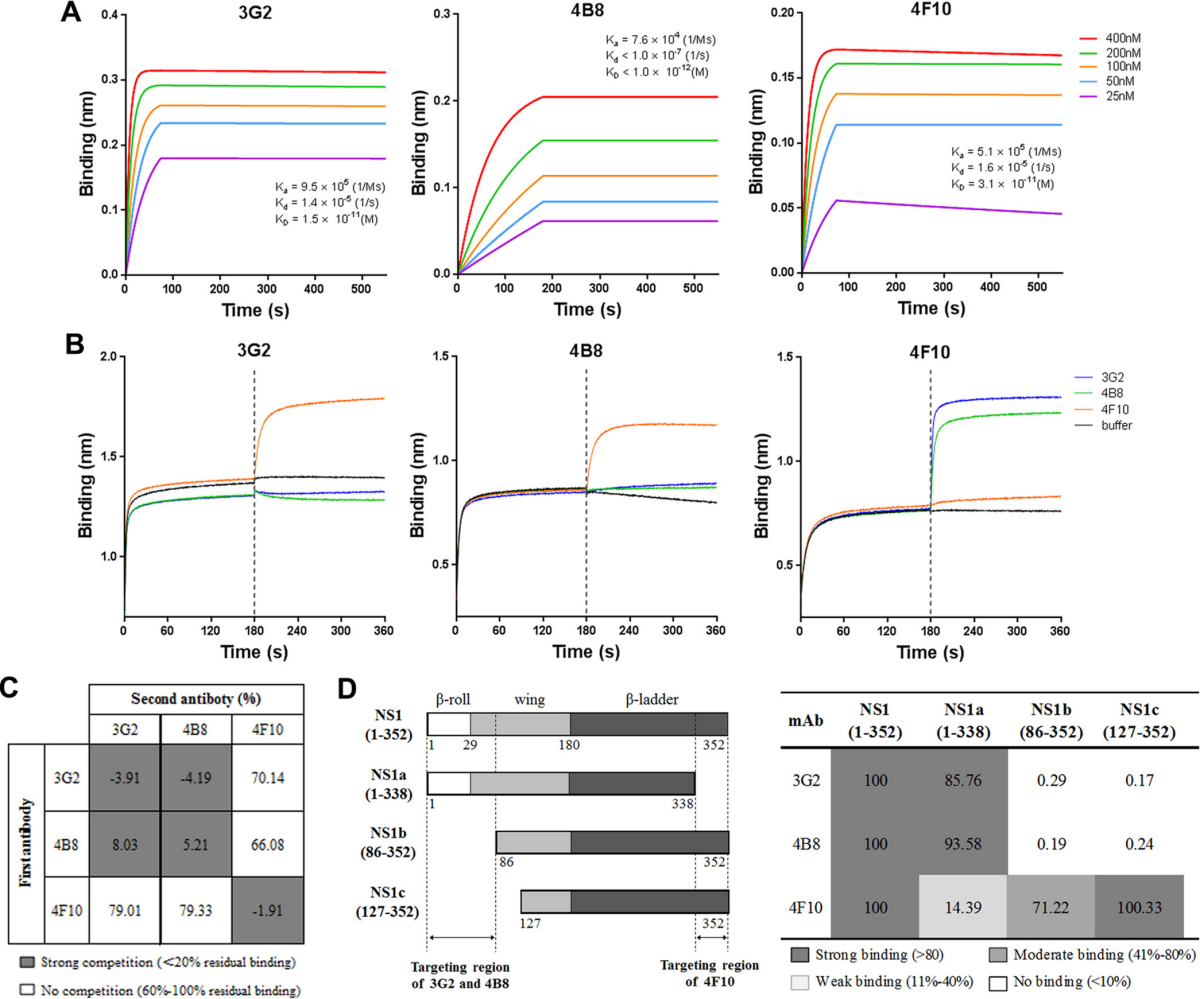
MAbs 3G2 and 4B8 recognize epitopes at the N-terminal region, whereas MAb 4F10 targets the C-terminal region of NS1. (A) Binding kinetics of 3G2 (left), 4B8 (middle), and 4F10 (right) with ZIKV NS1 protein were measured by BLI. The *K_a_*, *K_d_*, and *K_D_* values for each MAb are shown. (B) Competitive binding of 3G2, 4B8, and 4F10 against each other. (C) Competitive-binding analysis of NS1-targeted MAbs to ZIKV NS1. The numbers indicate the residual percent binding of the second antibody in the presence of the first antibody relative to the binding of the second antibody alone. (D) Schematic diagram of the full-length NS1 and the truncated mutants (left) and the binding intensity of MAbs 3G2, 4B8, and 4F10 to the three truncated mutants (right). The predicted binding sites of these MAbs are indicated. The relative binding of each MAb to the truncated mutants was calculated as the percentage of the optical density (OD) value from the binding with truncated mutants relative to the OD value of binding with the full-length NS1. Data are the representative results from three independent experiments. Each data point in panels C and D represents the mean from three technical replicates.

To determine the targeting regions of these MAbs, we first examined the kinetics of their binding to NS1 protein. All three MAbs exhibited high affinity toward NS1 (Fig. 7A). We then analyzed the ability of these MAbs to compete against each other for binding to NS1. MAbs 3G2 and 4B8 did not compete with MAb 4F10, but they competed with each other (Fig. 7B and C), suggesting that the binding sites of 3G2 and 4B8 either sterically overlap or are adjacent and that the binding of one MAb causes steric hindrance for the binding of the other MAb. This result implies that the targeting regions of MAbs 3G2 and 4B8 are different from that of MAb 4F10.

In an attempt to map the binding sites of these MAbs, we performed serial passage of ZIKV in the presence of each MAb and sequenced the viral genomes from passage 3 and passage 8. We did not observe any amino acid changes in NS1 during passages (see Fig. S6). In contrast, the E-targeted MAb 7B3 that recognized an epitope region covering residues T335, G337, E370, N371, and K394 (34) resulted in two amino acid changes (K251R and E370D) in passage 3 and four amino acid changes (K251R, G337A, E370D, and N371H) in passage 8 (see Fig. S7). This result implies that the selective force of NS1-targeted MAbs is not as strong as that of E-targeted MAbs or that amino acid changes in the epitopes of these NS1-targeted MAbs may exert massive selection pressure on ZIKV.

10.1128/mBio.03179-20.6 FIG S6 Alignment of the amino acid sequences of NS1 protein from different passages of ZIKV. ZIKV GZ02 strain was passaged on Vero cells 8 times in the presence of the three NS1-targeted MAbs, an E-targeted MAb (7B3), or PBS. The NS1-coding sequences were amplified from each passage and sequenced. The amino acid sequences of NS1 protein from each passage were aligned with that from the stocks. Download FIG S6, TIF file, 1.6 MB. Copyright © 2021 Yu et al. 2021 Yu et al. This content is distributed under the terms of the Creative Commons Attribution 4.0 International license.

10.1128/mBio.03179-20.7 FIG S7 Alignment of the amino acid sequences of E protein from different passages of ZIKV. ZIKV GZ02 strain was passaged similarly as described for Fig S6. The E-coding sequences were amplified from each passage and sequenced. The amino acid sequences of E protein from each passage were aligned with that from the stocks. Download FIG S7, TIF file, 2.3 MB. Copyright © 2021 Yu et al. 2021 Yu et al. This content is distributed under the terms of the Creative Commons Attribution 4.0 International license.

To further map the binding sites of NS1-targeted MAbs, we expressed three truncated NS1 fragments: NS1a (residues 1 to 338), NS1b (residues 86 to 352), and NS1c (residues 127 to 352). We tested the binding of each MAb to the truncated NS1 (Fig. 7D). MAbs 3G2 and 4B8 recognized the full-length NS1 and NS1a but not NS1b and NS1c, implying that the N-terminal region is critical for the binding of MAbs 3G2 and 4B8. In contrast, MAb 4F10 showed strong binding to full-length NS1, NS1b, and NS1c but showed weak binding to NS1a, suggesting that the C-terminal region is important for the binding of MAb 4F10 (Fig. 7D). Given that MAbs 3G2 and 4B8 but not 4F10 had both ADCC activity and direct inhibitory effects (Fig. 3), MAbs targeting different regions on NS1 may have different protective mechanisms against ZIKV infection.

## DISCUSSION

ZIKV has posed a significant burden to global public health due to its association with microcephaly in infants and its cocirculation with other flaviviruses, including DENV (2). Preventive and therapeutic approaches that suppress ZIKV disease but do not cause ADE of flavivirus infection are urgently needed. We report here that a subset of NS1-targeted MAbs inhibit ZIKV infection via both FcγR-dependent and -independent pathways without causing ADE of infection of either ZIKV or DENV. These merits not only result in effective protection against ZIKV pathogenicity but also provide additional virological benefits, as shown by the decreased viral loads in ZIKV-challenged neonatal mice that received MAbs 3G2 and 4B8 (Fig. 1 and 2). This expands our knowledge on the protective roles of NS1-targeted MAbs in addition to their previously proposed roles in mediating ADCC or complement activation (26, 35).

Several mechanisms of action have been described for antibodies targeting flaviviral NS1, such as blocking the endothelial permeability caused by NS1 protein (15), reducing the production of progeny virions through FcγR-dependent cytolysis of the infected cells (36, 37), or suppressing viral infection through both FcγR-dependent cytolysis and an unknown FcγR-independent pathway (38, 39). An earlier study showed that NS1-targeted antibodies protected against a lethal ZIKV infection in an FcγR-dependent manner (26). A recent study shows that the *in vivo* protective effects of NS1-targeted antibodies rely on the recognition of cell-surface NS1 and the engagement of Fc effector functions (35). Another study, however, suggests that NS1-targeted antibodies contribute to the control of ZIKV replication but cannot confer sufficient protection without T cell-mediated immunity, especially in interferon-α receptor knockout (IFNAR^−/−^) mice (8). Because IFNAR signaling may be dispensable for the production of antibody responses but may be important for executing ADCC activity (40), the lack of protection by NS1-targeted antibodies in IFNAR^−/−^ mice may be attributed to the inefficiency of ADCC activity or the relatively low concentration of protective antibodies. Our NS1-targeted MAbs exhibit comparable ADCC activity but show different protective efficacies in neonatal mice (Fig. 1 and 3), implying that the *in vivo* protection is not solely mediated via ADCC and that other pathways might be involved. We thus propose that there are at least two types of ZIKV NS1-targeted MAbs based on their mechanisms of action. One type of MAb, such as MAb 4F10 and the MAbs described in other studies, is able to trigger FcγR-mediated phagocytosis but cannot inhibit ZIKV infection without effector cells (Fig. 3 and 4). Therefore, the *in vivo* protective effects are mainly dependent on Fc-mediated effector functions (26, 37–39). MAbs 3G2 and 4B8 represent another type of MAb. This type of MAb not only destroys ZIKV-infected cells through ADCC but also inhibits ZIKV infection without the help of effector cells. MAbs 3G2 and 4B8, as well as their LALAPG variants, are able to reduce the viral loads in neonatal mice (Fig. 1, 2 and 5). Since MAb 3G2 has significantly better protective effects than MAb 4F10 regardless of the inoculation strategy used, an ideal NS1-targeted MAb should be able to trigger multiple protective pathways.

Our findings that some NS1-targeted MAbs exert a certain inhibitory effect on ZIKV infection may add to the understanding of their protective mechanisms. These MAbs are unlikely to block viral binding or entry, because NS1 proteins are not present on virions. Given that DENV NS1 has been shown to be endocytosed into host cells and enhance the early replication of DENV (41), ZIKV NS1 may have a similar function (42), and MAbs 3G2 and 4B8 may interrupt this function and impair viral replication (see Fig. S5 in the supplemental material). It has also been shown that flaviviral NS1 modulates the production of infectious viral particles (13, 43). We show that ZIKV release can be suppressed by MAbs 3G2 and 4B2 (Fig. S5), implying that NS1 protein may also be involved in the production of progeny ZIKV virions and that a proportion of NS1-targeted MAbs may be able to inhibit this process. The detailed molecular mechanisms through which MAbs 3G2 and 4B8 confer these inhibitory effects need to be clarified in future studies. Interestingly, we show that MAbs 3G2 and 4B8, as well as their LALAPG variants, significantly reduce the viral loads in neonatal brain. Under normal physiological conditions, the blood-brain barrier (BBB) restricts the access of IgG antibodies to the brain (44). However, these two MAbs may suppress ZIKV replication before it enters the brain, which reduces the influx of ZIKV across the BBB. Additionally, it is possible that these two MAbs are able to cross the BBB that has been compromised by ZIKV and confer protection in the brain (45). Another possible function of MAbs 3G2 and 4B8 is to disrupt the pathogenic roles of ZIKV NS1, which include increasing the permeability of brain endothelial cells and causing brain vascular leakage (20, 22, 46), and thus reduce the dissemination of ZIKV into neonatal brain. Animals that received MAbs 3G2 and 4B8 but not MAb 4F10 showed no obvious brain inflammation and abnormality (Fig. 1), also supporting that these MAbs may be able to reduce NS1 protein-mediated pathogenicity. Notably, NS1-targeted MAbs cannot select escape mutants during serial passage (Fig. S6 and S7). Because NS1 is a relatively well-conserved protein among mosquito-borne flaviviruses (47), an amino acid substitution in the epitopes of these MAbs may be detrimental to ZIKV. Another possibility is that the selection force of these MAbs may not be sufficient to create escape mutants. Hence, the application of these NS1-targeted MAbs is unlikely to generate ZIKV escape variants as the E-targeted antibodies do.

For the evaluation of NS1-targeted vaccines or MAbs, immunocompromised adult mice are frequently used (25–28). However, the immunocompromised status of these mice is achieved either by blockade of the interferon pathway or by knockout of key immune-related genes. This might not fully mimic the natural situation of susceptible hosts such as fetuses and newborns (48). Indeed, the vaccine candidate Ad2-prME that shows complete protection in adult mice only confers partial protection in a maternal immunization and neonatal challenge mouse model (29). The ZIKV-infected C57BL/6 neonatal mice may provide an alternative animal model for assessment of NS1-targeted MAbs. C57BL/6 neonatal mice have been reported to be susceptible to ZIKV infection and pathogenicity in several studies (29, 32). Multiple-dose inoculation with NS1-targeted MAbs, either the wild-type MAbs or the LALAPG variants, provided significantly better protection than the single-dose inoculation strategy (Fig. 1, 2, 5, and 6), indicating that a sustained high titer is required for NS1-targeted MAbs to confer protective effects in neonatal mice. The results of FNT with or without MAb overlay also support that NS1-targeted MAbs inhibit ZIKV infection only when they are present throughout the infection cycle (Fig. 3 and 4). Thus, a multiple-dose inoculation strategy is preferable for NS1-targeted MAbs to achieve significant protective efficacy.

The relationship between the location of the epitope and the protective efficacy of a ZIKV NS1-targeted MAb has not been reported. MAbs 3G2 and 4B8 recognize epitopes at the N-terminal region, whereas MAb 4F10 most likely targets the C-terminal region (Fig. 7). All the C57BL/6 neonatal mice that received MAb 3G2 but not those that received 4F10 survived a lethal dose of ZIKV infection and showed no obvious brain damage, implying that the region recognized by MAb 3G2 may contain a potential protective epitope (Fig. 1 and 2). MAbs directed to this type of epitope may have higher protective efficacy than MAbs targeting other epitopes, such as the one recognized by MAb 4F10. DENV NS1-targeted antibodies exhibit a similar pattern. Antibodies elicited by the NS1 protein with the C-terminal region deleted provide better protection against DENV infection than those elicited by the full-length NS1 (49). One MAb that targets the NS1 wing domain, which is located at the region adjacent to the N terminus of NS1, is protective against DENV infection in mice (50). Because the NS1-targeted MAbs reported in this study could not create escape mutants, resolving the structure of the NS1-MAb complex is needed to delineate the exact epitopes of MAbs 3G2 and 4B8.

In summary, we identify a subset of MAbs that target the N-terminal region of ZIKV NS1, which not only trigger ADCC but also inhibit ZIKV infection without effector cells. Compared to MAb 4F10 that targets the C-terminal region, these MAbs confer an additional virological benefit in a ZIKV-challenged neonatal mouse model. The existence of at least two subsets of ZIKV NS1-targeted MAbs and the association of their protective effects with the targeting region may provide insights for developing NS1-based prophylaxis and therapeutics against ZIKV infection.

## MATERIALS AND METHODS

### Cell lines.

African green monkey kidney cells (Vero, ATCC catalog number [cat. no.] CCL-81) and human chronic myelogenous leukemia cells K562 (ATCC cat. no. CCL-243) were grown in Dulbecco’s modified Eagle’s medium (DMEM; Gibco) supplemented with 10% fetal bovine serum (FBS) (Gibco) and antibiotics (100 U/ml penicillin and 100 μg/ml streptomycin; Gibco). Human peripheral blood mononuclear cells (PBMCs) were isolated from healthy blood donors and cultured in RPMI 1640 medium (HyClone) supplemented with 10% FBS. The use of human samples was reviewed and approved by the ethics committee of Guangzhou Eighth People’s Hospital. Informed consent was obtained from each of the blood donors. All cells were incubated in a humidified 37°C incubator (Thermo Fisher Scientific) with 5% CO_2_.

### Viruses.

Zika virus GZ02 strain (GenBank KX056898.1), dengue virus type 2 (GenBank AF038403), and dengue virus type 4 (GenBank AY947539) were propagated in Vero cells. The viral titers were titrated using plaque-forming assays on Vero cells, and the viruses were stored at −80°C until use.

### MAb production.

NS1-targeted MAbs (3G2, 4B8, and 4F10) and E-targeted MAbs (7B3 and 8D10) were isolated from two Chinese travelers with confirmed ZIKV infection and have been described elsewhere (31, 34). Fc mutations L234A, L235A, and P329G (LALAPG) were generated using PCR-based mutagenesis. The MAbs and the Fc-mutated variants were produced as follows. In brief, the plasmids encoding the heavy chain and light chain of each antibody were transiently cotransfected into HEK293-6E cells. Cells were then cultured in suspension in serum-free FreeStyle 293 expression medium (Thermo Fisher Scientific). Six days later, the supernatants of cell cultures were harvested and loaded onto protein A columns for purification. The concentration of purified antibody was measured using the *A*_280_ method.

### Mouse experiments.

Eight-week-old male and female C57BL/6 mice were purchased from Beijing Vital River Laboratory Animal Technology Co. Ltd. All animals were bred and housed in the animal experimental center of Guangzhou Institutes of Biomedicine and Health (GIBH). The experimental protocols were approved by the Institutional Animal Care and Use Committee of GIBH. All infection experiments were conducted under animal biosafety level 2 plus conditions.

One-day-old suckling mice were i.p. inoculated with ZIKV at 1.2 × 10^3^ PFU per mouse. At 1 h postinfection, NS1-targeted MAbs 3G2, 4B8, and 4F10 or their respective Fc-mutated variants were i.p. administered. MAb MR78 against Marburg virus was used as an irrelevant MAb control. For the multiple-inoculation strategy, the MAbs were inoculated at a dose of 20 μg per mouse on days 0, 3, 6, 9, and 12 postinfection. For the multiple-inoculation strategy, the MAbs were inoculated at a dose of 100 μg per mouse at 1 h postinfection. Body mass of each mouse was monitored daily. At 16 days postinfection, the surviving mice were euthanized. The brain tissues were harvested after the mice were sacrificed or after the mice had died due to ZIKV infection. The brain tissues were then subjected to examination of mass, viral load, and histological analysis.

### Neurological analysis.

On day 15 after ZIKV challenge, the neurological symptoms of the neonates were scored in a single-blinded manner (29). In brief, the neurological score of each limb was designated as follows: 0, no sign; 1, weakness or altered gait; 2, paresis; 3, full paralysis. The neurological score of the tail was designated as follows: 0, no sign; 1, half paralysis; 2, full paralysis. The score of a neonatal mouse was calculated as the sum of the scores from four limbs and the tail. Thus, the maximum score of an examined animal is 14. An animal that died from infection received a clinical score of 15.

### Histological analysis.

The neonatal brains were harvested at the time of autopsy, immediately fixed in 10% neutral buffered formalin for 7 days, and then transferred into 70% ethanol. Individual brain tissue was placed in processing cassettes, dehydrated through a serial alcohol gradient, and embedded in paraffin wax blocks. The 5-μm-thick tissue sections were dewaxed in xylene, rehydrated through decreasing concentrations of ethanol, and washed with PBS. The tissue sections were then stained with hematoxylin solution for 8 min and with eosin solution for 3 min. Finally, the sections were successively incubated with 70% ethanol for 20 s, 90% ethanol for 20 s, 100% ethanol for 1 min, and xylene for 3 min. The images were captured using the Motic VM V1 version 1.1 (Motic) real-time microscopic image acquisition system.

### Quantitative reverse transcription-PCR assay.

Genome copy number of ZIKV in the brain was measured using a quantitative reverse transcription-PCR assay (RT-qPCR) as described previously (29). In brief, total RNA was extracted from the neonatal brains using an RNeasy lipid tissue minikit (Qiagen) and was subjected to one-step RT-qPCR using a QuantiTect SYBR green RT-PCR kit (Qiagen) according to the manufacturer’s protocol. The primers were as follows: forward primer, 5′-TGGAGGCTGAGGAAGTTCTAG-3′, and reverse primer, 5′-CTTCACAACGCAATCATCTCCACTG-3′. Amplification was performed at 50°C for 30 min, 95°C for 10 min, and 45 cycles of 95°C for 30 s, 55°C for 30 s, and 72°C for 30 s, and a melting curve was produced at 65°C to 95°C with an increment of 0.5°C per cycle for 5 s. The standard curve was constructed with serial dilutions of ZIKV RNA fragments corresponding to the NS5 region generated by *in vitro* transcription. The viral loads were calculated as ZIKV genome copies per gram of tissue, and the detection limit was 1 × 10^4^ copies per gram tissue.

### Plaque-forming assay.

Mouse brains were homogenized using ceramic beads. Live ZIKV virions were measured via a plaque-forming assay. In brief, confluent Vero cells in 6-well plates were incubated with serial dilutions of the brain tissue homogenates in serum-free DMEM for 2 h followed by the addition of 1.2% Sepharose. After incubation for 4 days, cells were fixed with 10% buffered formalin and labeled with 3 μg/ml anti-ZIKV E MAb 8D10 (34). After being washed twice in PBS, cells were stained with horseradish peroxidase (HRP)-conjugated anti-human IgG antibodies (Proteintech). The plaques were developed with the AEC substrate set (BD Biosciences). Virus titers in the brains were calculated by dividing the plaque counts by the mass of homogenized brain tissues and expressed as PFU per gram.

### Immunofluorescence assay.

In brief, Vero cells were seeded into 24-well plates and infected with ZIKV at 2 PFU per cell. At 48 h after infection, cells were fixed with 4% paraformaldehyde and permeabilizated with 0.3% Triton X-100. Subsequently, cells were labeled with MAbs 3G2, 4B8, and 4F10, an E-targeted MAb 8D10, or an irrelevant MAb MR78. After washing 3 times with PBS containing 0.05% Tween 20 and 5% bovine serum albumin (BSA), cells were incubated with a fluorescein isothiocyanate (FITC)-conjugated goat anti-human IgG antibody (Beyotime) for 1.5 h. The cell nuclei were stained with 4′,6-diamidino-2-phenylindole (DAPI; Beyotime) at 37°C for 5 min. Fluorescence images were then captured by an Eclipse Ti-U (Nikon).

### Enzyme-linked immunosorbent assay.

Vero cells (2 × 10^4^ cells/well) were seeded into 96-well plates and infected with ZIKV at 4 × 10^4^ PFU/well. Two days later, the infected cells were fixed with ice-cold acetone at room temperature for 15 min. After blocking with PBS containing 5% nonfat milk for 1 h, the plates were washed with PBST (PBS supplemented with 0.05% Tween 20). ZIKV NS1-targeted MAbs and MAb MR78 were serially diluted, added to the plates, and incubated at 37°C for 2 h. The plates were then washed with PBST, HRP-conjugated anti-human IgG (Proteintech) was added, and the plates were incubated at 37°C for 1 h. Finally, the plates were developed using 3,3′,5′,5-tetramethylbenzidine (TMB) HRP substrate (KPL). The absorbance values were determined at 450 nm by the Synergy HT multi-mode microplate reader (BioTek Instruments).

### Flow cytometry-based neutralization test.

FNT was performed based on our previously reported method (29). For the FNT without MAb overlay, 2 × 10^4^ Vero cells were seeded into 96-well plates and cultured overnight. Serial dilutions of NS1-targeted MAbs or irrelevant MAb MR78 were mixed with ZIKV (4 × 10^4^ PFU/well), incubated at 37°C for 1 h, and inoculated onto Vero cells. After 2 h of infection, the infection mixtures were replaced with DMEM containing 2% FBS. At 4 days postinfection, the cells were fixed and permeabilized using BD Cytofix/Cytoperm according to the manufacturer’s protocol (BD Biosciences). Subsequently, the cells were labeled with an E-targeted anti-flavivirus monoclonal antibody, 4G2 (Millipore), stained with phycoerythrin (PE)-conjugated goat anti-mouse IgG (BioLegend), and analyzed on an Accuri C6 flow cytometer (BD Biosciences). Neutralization activity was calculated as the percent reduction in the E-positive cells relative to that for the virus-only control wells. The FNT with MAb overlay was performed similarly, except that the infection mixtures were not washed off; thus, the MAbs were present in the culture medium throughout the infection cycle. At 4 days after infection, the cells were examined as described in FNT without MAb overlay. The half-maximal inhibitory concentration titer (IC_50_) was calculated as the concentration of MAbs at which the E-positive cells was reduced by 50% relative to that in the virus-only control wells.

### Antibody-dependent cell-mediated cytotoxicity.

The ADCC assay was modified from a previously described method (26). In brief, 2 × 10^4^ Vero cells were seeded into 96-well plates and infected with a replication-incompetent adenovirus type 2 vector expressing GZ02 NS1 (Ad2-NS1) at 100 viral particles per cell. At 2 days postinfection, the infected Vero cells were incubated with serial dilutions of MAbs and used as target cells. Irrelevant MAb MR78 was also examined in parallel. Subsequently, human PBMCs were added to the culture mix at 2 × 10^5^ cells per well. ADCC was then allowed to occur over 8 h. Finally, the release of lactate dehydrogenase (LDH) was measured by an LDH cytotoxicity assay kit (Yeasen, China) as a surrogate marker of target cell lysis. The optical density values were determined at 490 nm by the Synergy HT multi-mode microplate reader (BioTek Instruments).

### Viral binding and entry assays.

The binding and entry assays were performed according to a previously described method (51). In brief, ZIKV (2 PFU per cell) was incubated with MAb 3G2, 4B8, or 4F10, an irrelevant MAb, MR78, at 100 μg/ml or PBS at 37°C for 2 h. The E-targeted MAb 7B3 (4 μg/ml) was also assessed as a positive control. After chilling on ice for 1 h, the mixtures were inoculated onto Vero cells and incubated at 4°C for 2 h. For the binding assay, the cells were thoroughly washed with ice-cold PBS and immediately subjected to RNA extraction using an RNeasy lipid tissue minikit (Qiagen). For the entry assay, cells were thoroughly washed with ice-cold PBS, resuspended, incubated at 37°C for another 2 h, and then subjected to RNA extraction. The genome copies of cell-bound or internalized viral particles were determined by RT-qPCR. The relative cell-bound virions were calculated as the ratio of genome copies of each MAb-treated group to those of the PBS-treated group and multiplied by 100. The relative internalized virions were calculated similarly.

### Viral infection assay.

In brief, Vero cells were infected with ZIKV at 2 PFU per cell. At 0, 6, 12, or 18 h after infection, cells were added with each NS1-targeted MAb or irrelevant MAb MR78 at 100 μg/ml. After 6 h of incubation, cells were rinsed with PBS three times, and the culture supernatants were replaced with fresh culture medium (see Fig. S5A in the supplemental material). At 24 h after infection, the genome copies of ZIKV in the cells and the culture supernatants were determined by RT-qPCR as described above. The relative genome copies were calculated as the ratio of absolute genome copies in each MAb-treated group to those in PBS-treated group and multiplied by 100.

### Antibody-dependent enhancement of infection.

ADE effects of NS1-targeted MAbs on ZIKV and DENV infection were measured using a flow cytometry-based method. In brief, serial dilutions of NS1-targeted MAbs were mixed with ZIKV (GZ02, 1 PFU per cell), DENV 2 (0.2 PFU per cell), or DENV 4 (0.2 PFU per cell) in RPMI 1640 medium at 37°C for 1 h. An E-targeted MAb (8D10) was also examined as a positive control. The mixtures were added to K562 cells in 96-well U-bottom plates. Two days later, cells were fixed, permeabilized using Cytofix/Cytoperm (BD Biosciences), and labeled with a mouse anti-flavivirus E antibody 4G2 (Millipore). The cells were then stained with PE-conjugated goat anti-mouse IgG (BioLegend) and analyzed with an Accuri C6 flow cytometer (BD Biosciences). The infection efficiency was calculated as the percentage of E-positive cells relative to the control.

### Dissociation constant determination.

Equilibrium dissociation constant (*K_D_*) values were determined by biolayer interferometry (BLI) using the Octet K2 system (ForteBio, Inc.). The tested antibody (0.3 μg/ml in buffer consisting of 10 mM PBS [pH 7.4], 0.1% BSA, and 0.02% Tween 20) was loaded onto the biosensor coated with protein A (ForteBio, Inc.) for 10 min. After sample equilibration in the buffer, the biosensor tips were immersed in the solutions including recombinant ZIKV NS1 protein (Sino Biological) at five concentrations (400 nM, 200 nM, 100 nM, 50 nM, and 25 nM) until the binding of the antibody and antigen was nearly saturated. After association, the biosensor tips were moved to the buffer for dissociation. The absorption rate constant (*K_a_*), dissociation constant (*K_d_*), and *K_D_* were calculated through global fit of the kinetic curves using Data Analysis 11.0.

### Antibody competition assay.

The competition assay for the MAbs was performed using the Octet K2 system (ForteBio, Inc.) based on BLI in an in-tandem orientation. In brief, biotinylated NS1 was immobilized on streptavidin-labeled biosensors (ForteBio, Inc.) for 2 min at a concentration of 10 μg/ml. The first antibody was then bound to NS1 under a saturation condition, followed by binding of the second antibody. The concentration of each antibody used was 34 μg/ml, and the assay was conducted in 1× kinetics buffer (ForteBio, Inc.). Residual binding was calculated as the percent binding of the second antibody in the presence of the first antibody relative to the binding of the second antibody alone. A reduction in residual binding of the secondary antibody to <20% due to the presence of the first antibody indicates strong competition for binding. A reduction in residual binding of the secondary antibody from 20% to 60% indicates intermediate competition for binding. A reduction in residual binding of the secondary antibody to >60% indicates weak or no competition for binding.

### Truncated NS1 proteins and epitope analysis.

Based on a previously described expression plasmid for full-length NS1 (GenBank KY888678) (31), three plasmids expressing NS1a (residues 1 to 338), NS1b (residues 86 to 352), and NS1c (residues 127 to 352), each fused with a D7 tag at the C terminus, were constructed. The proteins were produced in HEK293T cells by transient transfection. Epitope analysis was performed using an enzyme-linked immunosorbent assay (ELISA) (31). In brief, truncated and full-length NS1 proteins were captured on 96-well ELISA plates that were precoated with an anti-D7 antibody (D7324; Cliniqa). MAbs 3G2, 4B8, and 4F10 were serially diluted and added to the plates in duplicates to recognize NS1 proteins. Binding activity was detected by HRP-conjugated goat anti-human IgG and developed by TMB substrate. After deduction of the background, the ratio between the optical density (OD) value obtained from the truncated NS1 and the OD value obtained from the full-length NS1 was calculated. The binding strength was designated as follows: >80%, strong binding; 40% to 80%, intermediate binding; 10% to 39%, weak binding; <10%, no binding.

### Statistical analysis.

Flow cytometry data were analyzed using FlowJo version 10 software (Tree Star Inc.). Comparisons of the body mass, brain mass, neurological symptoms scores, and the viral loads among different groups were conducted by one-way analysis of variance (ANOVA) and Tukey’s multiple-comparison test. Comparison of the survival rates was performed using the log rank test. The statistical analyses were computed with GraphPad Prism version 7 (GraphPad Software), and *P* values of less than 0.05 were considered statistically significant. Data graphs were generated using GraphPad Prism version 7 (GraphPad Software). Illustrations were generated using Microsoft PowerPoint version 2010 (Microsoft). Figures were created using Photoshop version CS2 (Adobe Systems Inc.).

### Data availability.

The published article includes all data analyzed for this study.
