# Supplementary figures and images for: Monoclonal Antibodies against Zika Virus NS1 Protein Confer Protection via Fcγ Receptor-Dependent and -Independent Pathways
Source: mBio. 2021 Feb 9;12(1):e03179-20. doi: 10.1128/mBio.03179-20 (PMC7885117; doi:10.1128/mBio.03179-20)

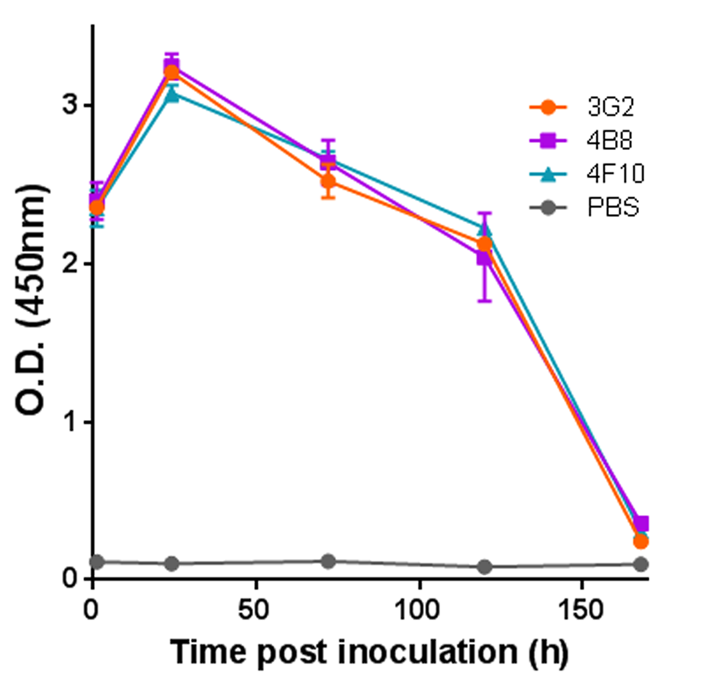

Supplement: FIG S1 [file mBio.03179-20-sf001.tif]

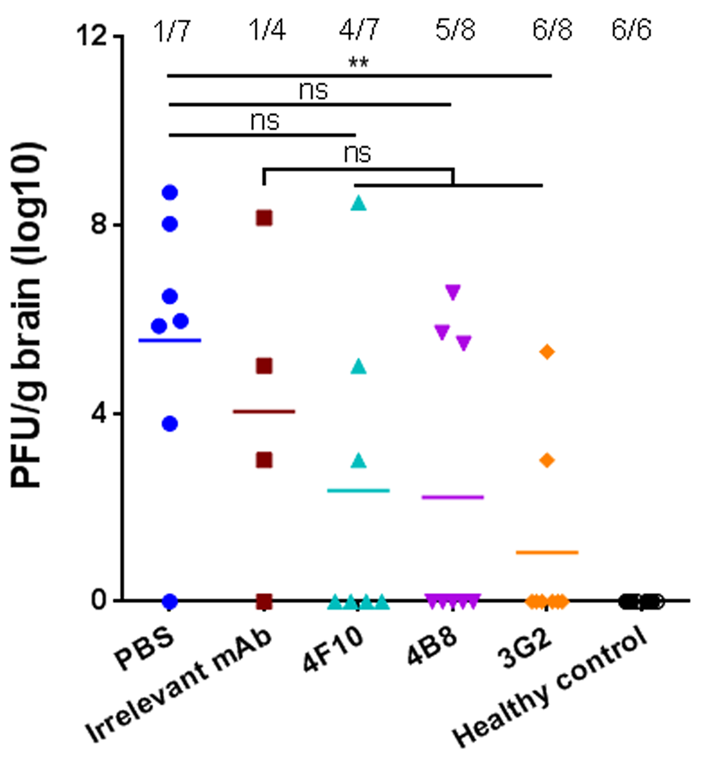

Supplement: FIG S2 [file mBio.03179-20-sf002.tif]

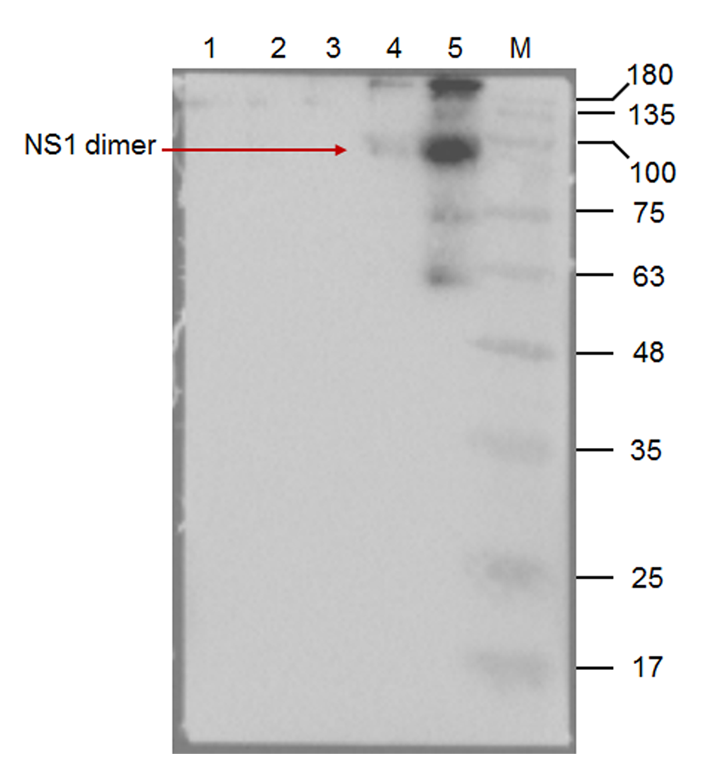

Supplement: FIG S3 [file mBio.03179-20-sf003.tif]

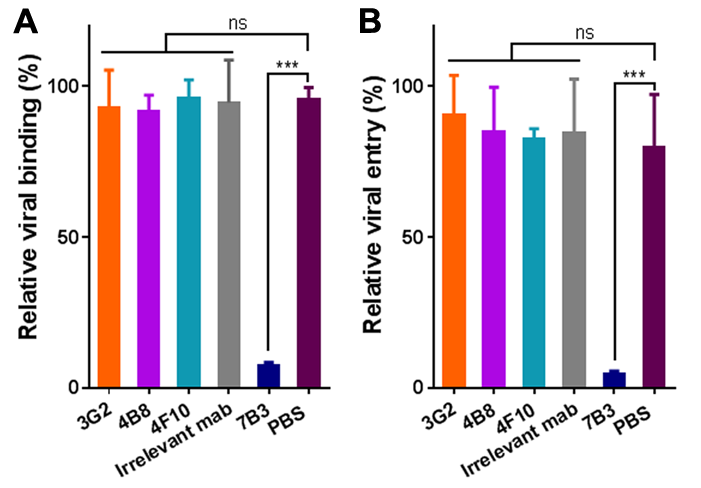

Supplement: FIG S4 [file mBio.03179-20-sf004.tif]

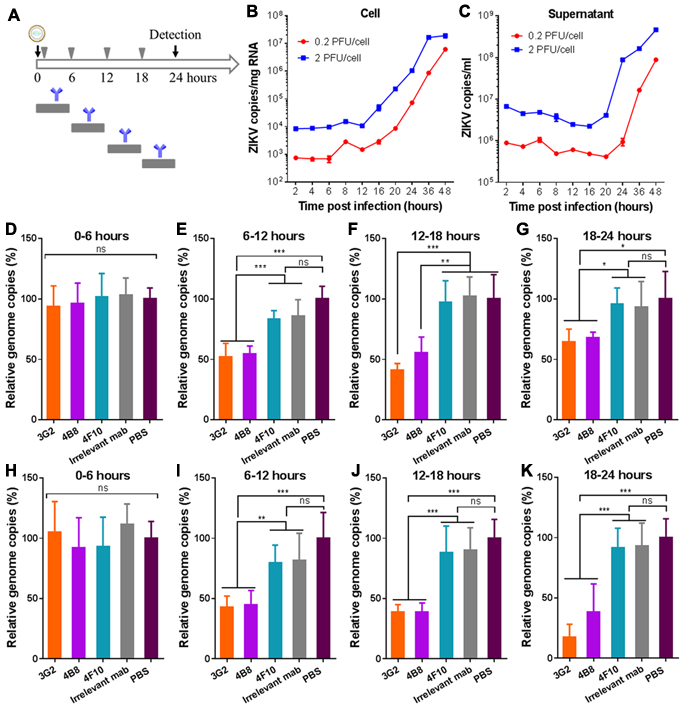

Supplement: FIG S5 [file mBio.03179-20-sf005.tif]

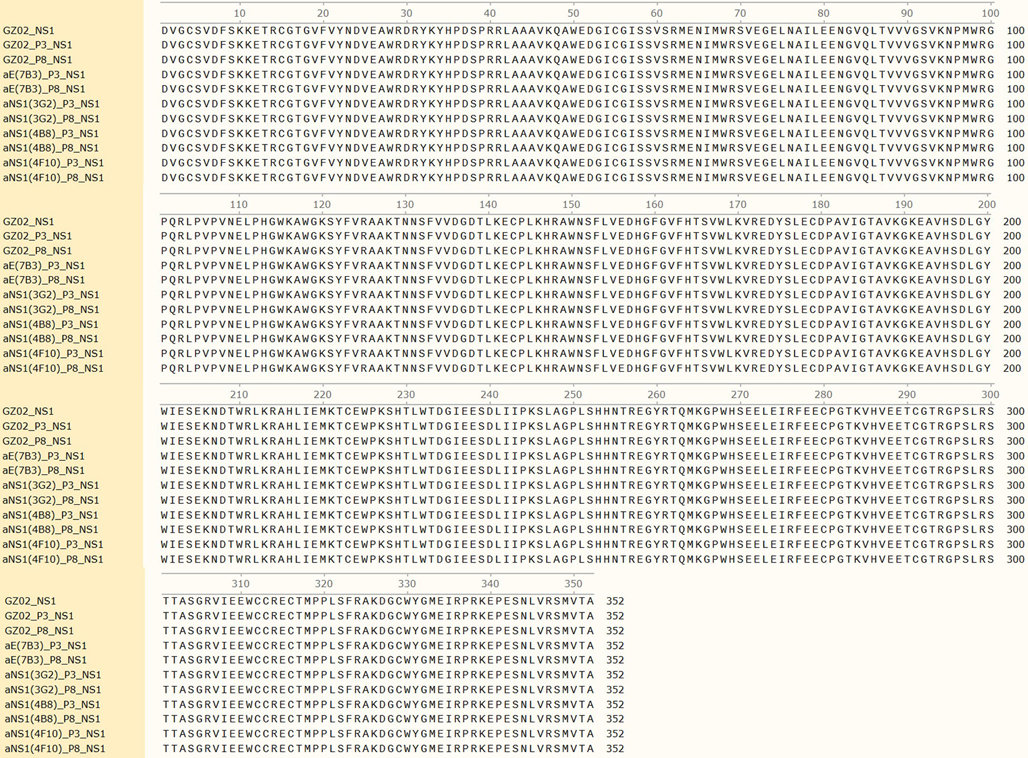

Supplement: FIG S6 [file mBio.03179-20-sf006.tif]

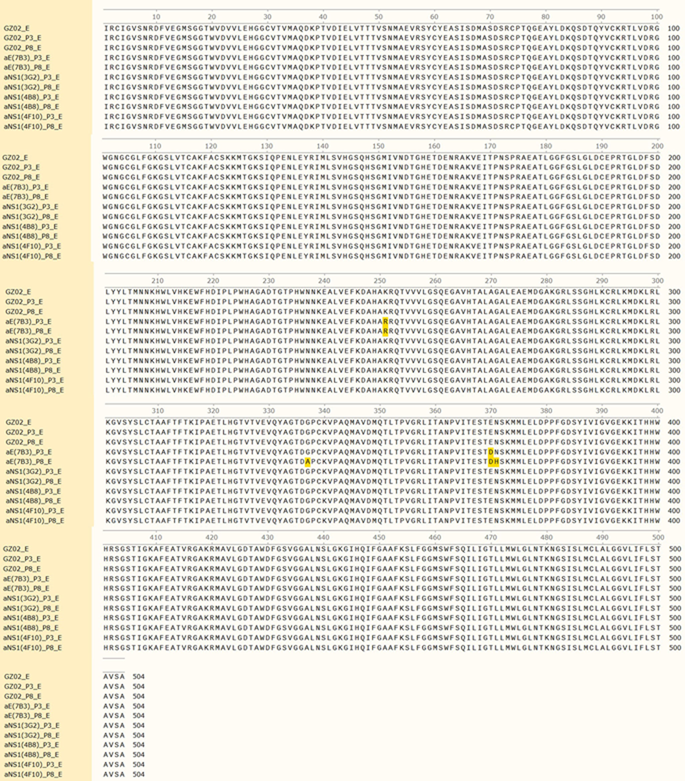

Supplement: FIG S7 [file mBio.03179-20-sf007.tif]
